# Defect Identification of Composite Insulator Based on Infrared Image

**DOI:** 10.3390/polym14132620

**Published:** 2022-06-28

**Authors:** Zhongyuan Zhang, Qilin Huang, Jianghai Geng, Qiyu Liu, Simin Zhang

**Affiliations:** Hebei Key Laboratory of Power Transmission Equipment Security Defense, North China Electric Power University, Baoding 071003, China; hvzzy_01@163.com (Z.Z.); dhuangqilin@163.com (Q.H.); dliuqiyu@163.com (Q.L.); 220192213164@ncepu.edu.cn (S.Z.)

**Keywords:** composite insulator, temperature rise, defect, infrared detection

## Abstract

The deterioration of a composite insulator’s mandrel will cause a serious power-grid accident, so it is necessary to find the defect as soon as possible. Infrared imaging is an effective means to detect mandrel-deterioration defects, but sheath-aging defects will cause trouble to screen mandrel deterioration. Therefore, it is urgent to distinguish the two defects. In this paper, two composite insulators that are out of service with different heating characteristics are tested and analyzed, and the temperature-rise characteristics are analyzed by building a simulation model. The results show that the temperature rise of composite insulators with oxidative decomposition of epoxy resin is large, and the range usually extends from the hardware of the high-voltage side to several umbrella skirts. The temperature rise caused by the composite insulator with the fiber completely wrapped by epoxy resin is small, which mainly occurs from the hardware to the first umbrella skirt. The simulation model constructed in this paper is consistent with the experimental results, which verifies the accuracy of the model. The model can distinguish the mandrel-deterioration defect and sheath-aging defect, and has guiding significance and important reference value for the detection rate of the mandrel-deterioration composite insulator and ensuring the safe and stable operation of the power grid.

## 1. Introduction

Composite insulators have the advantages of small mass, small volume, low installation difficulty and strong pollution resistance [1,2,3]. They have been widely used in 35kV-and-above overhead transmission lines of China’s power system since the 1980s [4,5]. To date, the number of composite insulators has exceeded 40 million, accounting for more than 45.5% of the total number of insulators [6,7,8].

However, the mandrel, the main component of the composite insulator, is made of organic synthetic materials, which have been affected by complex operating environmental conditions for a long time, resulting in deterioration defects of the mandrel [9,10]. These defects often lead to serious power-grid accidents, such as insulator breakage, wire dropping, short-circuit tripping, etc., which seriously endanger the stable operation of the power grid. Although the defects are hidden, they are often accompanied by an abnormal temperature rise. Therefore, the deterioration defects of a composite insulator’s mandrel can be effectively detected by means of infrared thermography [11,12]. However, the reason for the abnormal temperature rise of the composite insulator is not only the deterioration defect of the mandrel. The aging and moisture of the sheath will also cause the abnormal heating of the composite insulator [13,14], but this defect will not cause the fracture of the composite insulator and will not seriously endanger the stable operation of the power grid. Therefore, it is not necessary to replace it immediately. To sum up, only using the existence of abnormal temperature rise as the judgment basis for mandrel-deterioration defects will cause the possibility of false detection and missed detection, which will affect the economic and stable operation of the power grid. Therefore, it is particularly important to distinguish the heating characteristics of mandrel-deterioration defects and sheath-aging defects to improve the detection rate of mandrel deterioration and ensure the safe and stable operation of the power grid.

Literature [14,15,16] analyzed the microcharacteristics of a composite insulator’s sheath after aging via static contact-angle method and infrared spectrum-analysis method. Combined with infrared-imaging technology, it was found that a large number of microcracks, micropores and other microdefects were formed on the surface layer of the sheath after aging. In a high-humidity environment, moisture intruded into the microdefects of the sheath, causing abnormal heating of the composite insulator. In reference [17,18,19], abnormal-heating composite insulators were found during line inspection by means of infrared thermography. By means of scanning electron microscope, infrared spectrum, broadband dielectric spectrometer and other research methods, it was concluded that the abnormal heating of a composite insulator is caused by the deterioration defect of the mandrel, and the dielectric constant and the tangent of the dielectric loss angle (tan*δ*) of the mandrel at the deterioration point increased significantly. Reference [20] shows that the temperature rise of a composite insulator comes from the dielectric loss of its material. According to the simulation analysis of short composite insulator samples in document [21], it is concluded that the characteristics of composite insulators’ temperature rise are different under different degradation states or defect types.

The above methods and conclusions provide a reference for studying the heating characteristics of different defects of composite insulators, but the following aspects still need to be further improved. To begin with, most of the research focuses on the microscopic changes of composite insulator defects; infrared is only an observation means, and there is little research on the heating characteristics. Moreover, the above research assumes that the change in short sample is consistent with that of a full-size composite insulator. However, when analyzing the short sample, it is generally considered that the short sample is defective or normal as a whole, while the actual composite insulator will only have local defects. The correspondence between the two heating characteristics still needs to be further discussed.

In view of this, this paper tests and analyzes two composite insulators that are out of service with different abnormal-heating characteristics, builds a simulation model by using the finite-element method, analyzes the temperature-rise characteristics, and obtains the heating causes of the two abnormal-heating composite insulators. By comparing the simulation data with the measured data, the correctness and rationality of the simulation model are verified. The simulation model of composite insulators with different defects constructed in this paper has guiding significance and important reference value for revealing the temperature-rise characteristics of composite insulators with different defects.

## 2. Materials and Methods

### 2.1. Test Plan of the Temperature Rise

#### 2.1.1. Sample Selection

In this paper, an artificial climate chamber is built to carry out infrared temperature-rise test on a batch of 220 kV composite insulator samples that are out of service from Hainan. These composite insulator samples are tested as received.

#### 2.1.2. Test Platform and Layout

**Figure 1:**
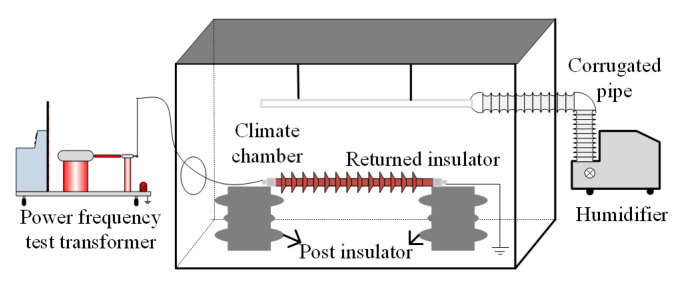
Temperature-rise test platform.

The test layout is shown in Figure 1. The size of the climate chamber is 4 m × 4 m × 3 m. The 220 kV composite insulators are arranged horizontally. The high-voltage terminal on the left side is connected to a power supply with test voltage of 140 kV, and the low-voltage terminal on the right side is grounded. The humidity is controlled within 95% by a humidifier, and the ambient temperature varies within ±1 °C during the test. An infrared imager FLIR T1040 (FLIR Systems Inc., Niceville, FL, USA) is used to record the temperature rise of the test objects. Referring to the high-humidity environment in Hainan, the temperature is set to 10 °C and the humidity is 75%.

### 2.2. Test of the Composite Insulator with Abnormal Temperature Rise

#### 2.2.1. Sample Selection

The sample with abnormal temperature rise in the above experiment is selected. In this section, the heating causes are analyzed through different tests.

#### 2.2.2. Visual Inspection and SEM Analysis

Firstly, the appearance of composite insulators with different heating characteristics is analyzed from the macro view to observe whether there are differences in appearance; Secondly, the mandrel-deterioration degree of the composite insulator with abnormal temperature rise is observed by scanning electron microscope (NOVA NANO SEM 230, USA). The samples are the mandrel at the abnormal heating part of the composite insulators, and these samples do not need to be pretreated.

#### 2.2.3. Thermogravimetric Analysis

Because glass fiber has the characteristics of high melting point (1600 °C), nonoxidation and noncombustion, for the material of the composite insulator’s mandrel, the pyrolysis of epoxy-resin matrix is the main cause of thermal weight loss of mandrel’s material below 1000 °C. Therefore, Thermogravimetric Analysis (TGA, PerkinElmer-STA6000, Waltham, MA, USA) can analyze the content of epoxy and fiber. The samples are the mandrel of the abnormal heating part of the composite insulator, and the normal composite insulator’s mandrel is used as the control. All these samples do not need to be pretreated. The test conditions are as follows: the heating range is from 50 °C to 800 °C, the heating rate is 10 °C/min, and the shielding gas is N_2_.

#### 2.2.4. Test of Dielectric Characteristic

The temperature rise of the composite insulator comes from the dielectric loss of its material, and the power loss per unit volume of the medium can be expressed as

(1) $$p=EJ_{r}$$

In the formula, *E* is the electric field strength, *J*_r_ is the active current density in the medium and the ratio of it to the reactive current density *J*_c_ in the medium is the tan*δ*, which is expressed by Formula (2):

(2) $$\tan\delta =J_{r}/J_{c}$$

The relationship between the reactive current density *J*_c_ and the electric field intensity *E* is shown in Formula (3):

(3) $$J_{c}=E\omega \varepsilon_{0}\varepsilon_{r}$$

In the formula, *E* is the electric field strength; *ω* is the angular frequency; *ε*_0_ is the vacuum permittivity, its value is 8.854 × 10^−12^ F/*m*; and *ε*_r_ is the relative permittivity.

According to Equations (2) and (3), it can be known that the active current density has a linear relationship with the relative permittivity and tan*δ*. The active current density increases with the increase in the relative permittivity and tan*δ*. Substituting Equations (2) and (3) into Equation (1), we can obtain:

(4) $$p=E^{2}\omega \varepsilon_{0}\varepsilon_{r}\tan\delta$$

Under the action of 50 Hz power frequency voltage, the angular frequency is constant, and the power loss is proportional to the relative dielectric constant and tan*δ*. Therefore, it is necessary to further analyze the relative dielectric constant and tan*δ* of the abnormal-heating samples.

Samples are collected from different positions of the abnormal heating insulator samples, such as the heating and nonheating parts of the samples, then they are put in a constant-humidity incubator and dried at 75% ambient humidity for 48 h. The relative dielectric constant and dielectric loss factor are measured by the tester of dielectric constant and dielectric loss (Shanghai Yanggao Electric Co., Ltd., Shanghai, China).

### 2.3. Simulation-Model Analysis

Aiming at the above-mentioned typical samples of composite insulators, this paper uses the finite-element simulation software COMSOL Multiphysics (COMSOL Inc., Stockholm, Sweden) to establish a three-dimensional model of composite insulators, simulate the heating phenomenon of the abnormal heating samples, and compare the temperature rise and temperature-rise distribution of the composite insulators.

#### 2.3.1. Model Parameters

The three-dimensional model is constructed with reference to the FXBW-220/100 model composite insulator parameters, as shown in Table 1.

**Figure 2:**
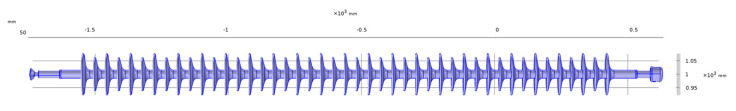
Full-size composite insulator model.

The composite insulator model is mainly composed of hardware, mandrel and sheath. The mandrel is made of glass-fiber-reinforced plastic (GFRP), and the sheath is made of silicone rubber. The three-dimensional model of the 220 kV composite insulator is shown in Figure 2.

#### 2.3.2. Model of the Heat Transfer

Heat transfer is mainly divided into heat conduction, heat convection and heat radiation. Since the heat radiation is relatively small compared to the heat conduction and heat convection, the heat-transfer model constructed in this paper only considers heat conduction and heat convection. The correlation coefficient of each material is shown in Table 2.

Thermal convection mainly refers to the phenomenon that heat is released from higher temperature to lower temperature during the flow of fluid (including liquid and gas). It can simulate the heat-transfer mode between the surface of the composite insulator and air. The formula is:

(5) $$q=h\left(T_{2}-T_{f}\right)$$

where *h* is the surface heat transfer; the unit is W/(m^2^·K). *T*_2_ and *T*_f_ represent the surface temperature and ambient temperature of composite insulator, respectively.

Heat conduction is a macroscopic phenomenon of heat transfer from high temperature to low temperature caused by the thermal motion and interaction of molecules or atoms constituting the system. It can simulate the heat-transfer mode between different materials of composite insulator. Its formula is

(6) $$q=-k\frac{dT}{\;dx}$$

where *q* is the heat-flux density of heat conduction, the unit is W/m^2^. *k* is the thermal conductivity, which reflects the thermal conductivity of the material, the unit is W/(m·K). d*T*/d*x* is the temperature gradient, the unit is k/m.

## 3. Results

### 3.1. Analysis of Test Results of the Temperature Rise

**Figure 3:**
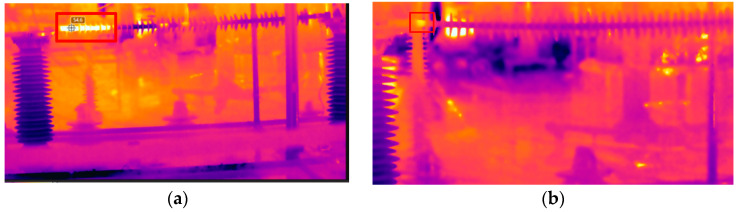
(**a**) Test object #1’s infrared image; (**b**) test object #2’s infrared image.

The test finds that two of the insulators have abnormal heating. The infrared images are shown in Figure 3. The maximum temperature rise of #1 is 40 °C, and the range of abnormal heating extends from the hardware of the high-voltage side to the seventh umbrella skirt. While the maximum temperature rise of #2 is 2 °C, the range of abnormal heating extends from the hardware to the first umbrella skirt, which is smaller than #1. Through the infrared image, it can be found that the two composite insulators have different heating characteristics; their temperature rise and the range of abnormal heating are different. It is preliminarily analyzed that the two composite insulators have different defects.

### 3.2. Analysis of Test Results of the Composite Insulator with Abnormal Temperature Rise

#### 3.2.1. Visual Inspection and SEM Analysis

**Figure 4:**
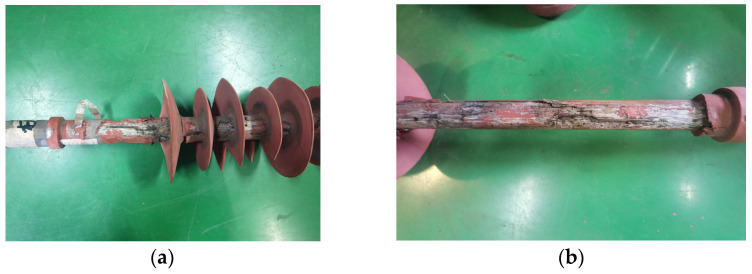
#1’s appearance and interior deterioration. (**a**) #1’s appearance; (**b**) without the skirts.

**Figure 5:**
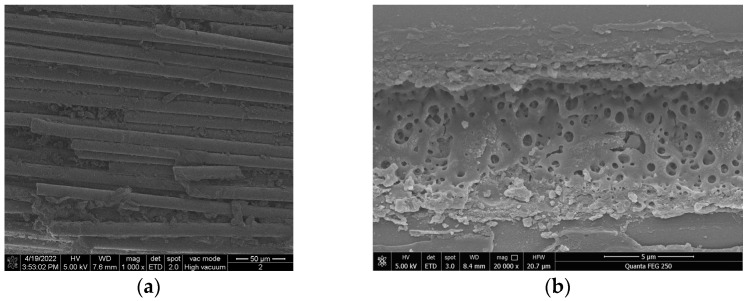
Micromorphology of #1’s mandrel. (**a**) Fiber exposed; (**b**) melting and degradation of epoxy resin.

**Figure 6:**
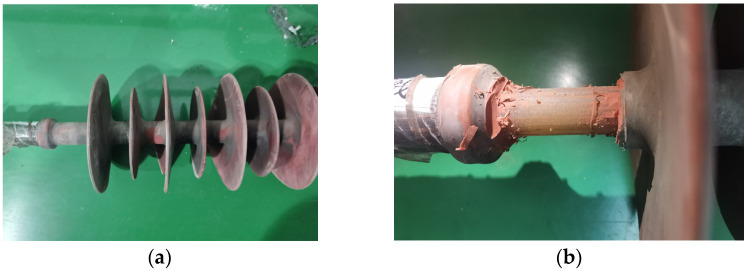
#2’s appearance and its mandrel. (**a**) #2’s appearance; (**b**) without the skirts.

**Figure 7:**
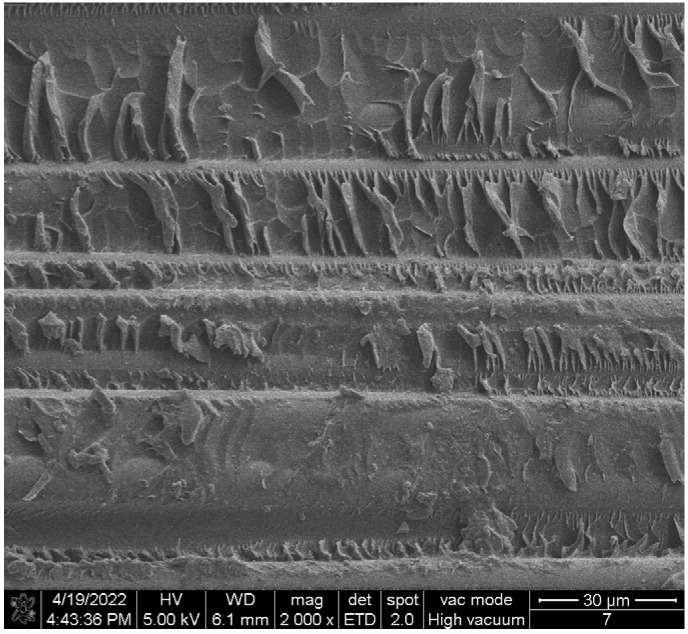
Micromorphology of #2’s mandrel.

In view of the abnormal-heating phenomenon of the two composite insulators, the appearance inspection and anatomical analysis of the two composite insulators are further carried out. The analysis conclusions are as follows:

(1) The sheath surface of composite insulator #1 is partially cracked, and the deterioration of the mandrel can be seen at the cracked part of the sheath. After the silicone rubber is removed, it can be seen that the mandrel of this insulator is like rotten wood, and the surface is blackened after the mandrel is carbonized, especially from the hardware of the high-voltage side to the seventh umbrella, as shown in Figure 4. The deterioration part of #1’s mandrel was observed by scanning electron microscope (SEM), and the results are shown in Figure 5. It can be seen from the figure that the glass fiber of the mandrel at this part is obviously exposed, and the epoxy resin is seriously damaged and oxidatively decomposed. The above results indicate that there is serious deterioration of #1’s mandrel, which may be the reason for its abnormal temperature rise.
(2) For test object #2, only the surface of the sheath is aged, the color is dark, and the toughness is reduced, as shown in Figure 6. However, there is no debonding phenomenon between the mandrel and the sheath, and the mandrel does not show obvious signs of deterioration. The abnormal heating part of #2’s mandrel was observed by scanning electron microscope (SEM), and the results are shown in Figure 7. It can be seen from the figure that the epoxy-resin filling of the mandrel in this part is relatively complete, there is no obvious degradation trace, and the glass fiber is basically intact. The above results indicate that the mandrel of #2 has no deterioration phenomenon. The deterioration of the mandrel may not be the cause of its abnormal temperature rise. Due to the aging phenomenon of the sheath, we guess that the aging of the sheath is the reason for the abnormal temperature rise.

#### 3.2.2. Thermogravimetric Analysis

**Figure 8:**
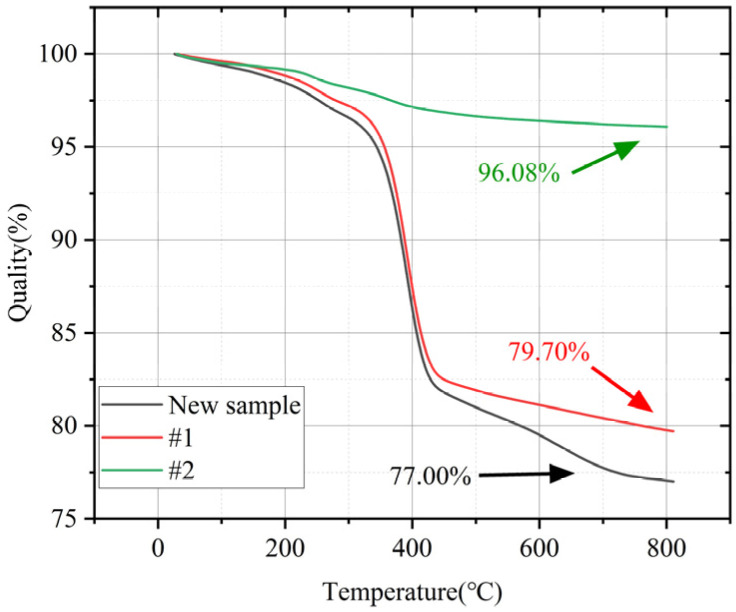
TGA curve of #1, #2.

The results are shown in Figure 8. It can be seen from the figure that the epoxy matrix begins to degrade at about 350 °C, and the degradation process ends at 430 °C. The weight-change rate of #2 and the new sample in this temperature range is 0.14%/°C, while the weight-change rate of #1 is 0.009%/°C. When the temperature reaches 800 °C, the epoxy resin is decomposed and the remaining component is glass fiber. Therefore, the glass fiber content in the new sample, #2 and #1 are 77.00%, 79.70% and 96.08%, respectively; the content of epoxy resin is 23.00%, 20.3% and 3.92%. Meanwhile, the mandrel of composite insulator #1 has obvious degradation of epoxy resin compared with the new sample, while the mandrel of composite insulator #2 has no obvious change. It is preliminarily guessed that #2 has no mandrel defect, while #1 has mandrel defect.

To sum up, the two composite insulators with different heating characteristics have obvious differences in appearance and component content. The mandrel of #1 has deterioration phenomenon, while the mandrel of #2 is complete and has no obvious signs of deterioration. It is preliminarily guessed that the abnormal heating of the two composite insulators is caused by different defects.

#### 3.2.3. Analysis of Test Results of Dielectric Characteristic

The dielectric characteristics of two test objects with abnormal heating are tested, and the parameters are shown in Table 3.

It can be seen from Table 3 that for sample #1, the relative permittivity and tan*δ* of the heat-generating part have a larger increase than those of the unheated part, and the relative dielectric constant of the mandrel in the heat-generating part is the unheated part 4.94 times, the tan*δ* value is 23.68 times that of the unheated part. Compared with the change of the mandrel, the change of the electrical parameters of the sheath at the heating part is small. The relative dielectric constant is 1.33 times that of the unheated part, and the tan*δ* is 3.22 times that of the unheated part. The reason for the analysis is that the sheath is cracked. In a high-humidity environment, water is more likely to enter the mandrel, and water molecules act as polar molecules to increase the relative permittivity and tan*δ* of the material.

For test sample #2, the sheath of the heat-generating part of the high-voltage side has a certain degree of increase compared with the rest of the part, the relative permittivity is 1.11 times that of the unheated part, and the tan*δ* value is 2 times that of the unheated part. The reason is that the electric field strength at the location of the high-voltage side is the largest, which makes it easy to accumulate moisture and contamination, and then makes the sheath at the high-voltage side the most serious-aging [15]. After the aging of the sheath, a large number of microcracks, micropores and other microdefects are formed on the surface layer. In a high-humidity environment, the intrusion of water into the microdefects of the sheath causes an increase in the dielectric constant and tan*δ*. However, because the sheath has good encapsulation on the mandrel, it is not easy for moisture to enter the mandrel.

The above test results can show that the electrical parameters (relative dielectric constant and tan*δ*) of the abnormal heating part have a certain degree of increase compared with the nonheating part, which proves that the abnormal temperature rise of the two composite insulators is related to the change in the electrical parameters of the material. The change in electrical parameters of the deteriorated mandrel of sample #1 is more significant than that of the sheath, and it is preliminarily determined that the abnormal temperature rise of #1 is caused by the mandrel, while the abnormal temperature rise of #2 is caused by the sheath.

### 3.3. Analysis of the Results of Simulation Model

#### 3.3.1. Effect of Mandrel Deterioration on Temperature Rise

**Figure 9:**
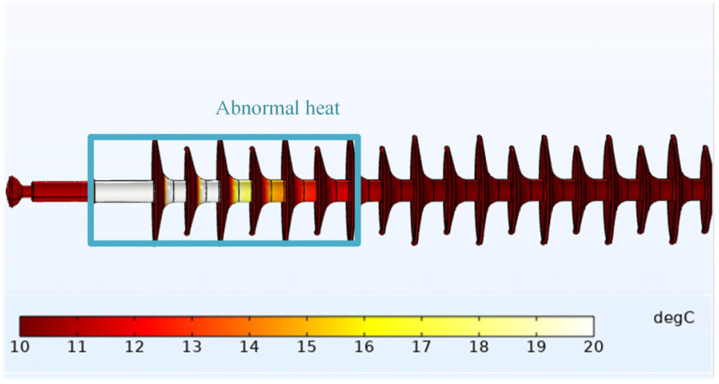
Simulation diagram of #1’s temperature rise.

Assuming that the ambient temperature and composite insulator’s temperature at the initial time are both 10 °C, the temperature distribution of composite insulator #1 after 60 min is shown in Figure 9.

From the simulation diagram of temperature rise, it can be preliminarily seen that there is a serious heating phenomenon from the high-voltage side to the seventh umbrella of #1. The maximum temperature of #1 can reach 55 °C, and the temperature rise is 45 °C, which is located at the high-voltage side.

**Figure 10:**
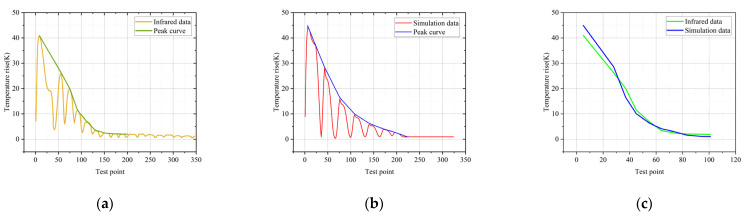
(**a**) Infrared temperature-rise curve of #1; (**b**) simulated temperature-rise curve of #1; (**c**) peak curve comparison diagram of #1.

In order to verify the correctness of the simulation, the infrared spectrum is extracted and compared with the temperature simulation results, as shown in Figure 10.

Figure 10a shows the temperature-rise data extracted from the infrared image in Figure 3a, and Figure 10b shows the temperature-rise data extracted from the simulation image in Figure 9. The temperature-rise curves in both figures start from the high-voltage side of the composite insulator, and the abscissa is the serial number of the temperature test points along the axis of insulator. The parts corresponding to the wave peaks are the sheath part between the umbrellas, and the part corresponding to the wave trough is the umbrella skirt. The reason why there is no obvious temperature rise at the umbrella skirt is that the electric field intensity, current density and loss power at the umbrella skirt are very low [20]. It can be seen from the figure that the maximum temperature rise is at the high-voltage side, and the temperature gradually decreases along the axis of the composite insulator. The abnormal temperature rise occurs from the hardware of the high-voltage side to the seventh umbrella skirt, and this range corresponds to the deterioration part described above. Compare the peak curve of Figure 10a with that of Figure 10b, as shown in Figure 10c.

It can be seen that the simulation results are consistent with the change trend of the actual infrared data. The temperature rise of the high-voltage side is the highest, and the temperature gradually decreases along the axis of the composite insulator. These characteristics of the simulation results are completely consistent with the infrared data. The Pearson correlation coefficient is 0.99286 and the *p*-value is 1.13 × 10^−8^, which indicates that the infrared data have a strong correlation with the simulation data. The accuracy of the simulation model is illustrated.

**Figure 11:**
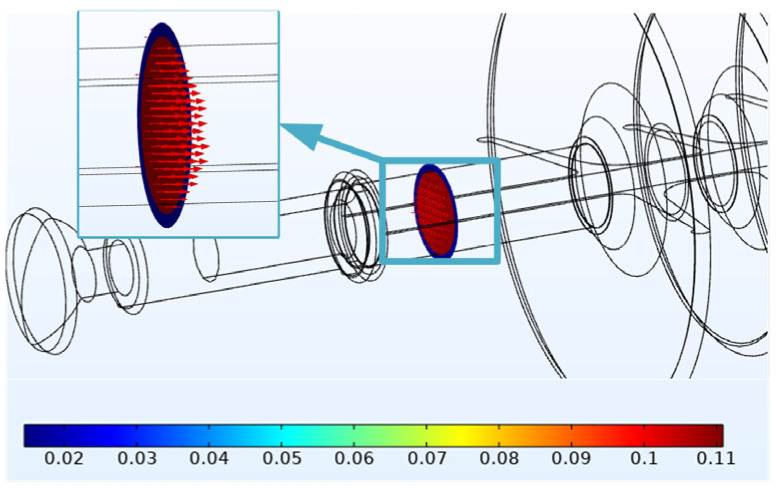
Current-density distribution of #1.

In order to explore the heating source of #1, the cross-sectional view of the high-voltage side with abnormal heating is further extracted for current-density analysis, as shown in Figure 11.

When t = 60 min, the current density inside the mandrel is 0.11 A/m^2^ and the current density inside the sheath is 1.5 × 10^−2^ A/m^2^. The results show that the leakage current mainly flows from the mandrel. According to Formula (1), we can know the corresponding relationships among the loss power, the electric field strength and the current density in the unit volume medium.

When the electric field intensity is the same, the loss power with high current density is greater, and the resulting temperature rise is also higher. In the section where the electric field strength is 2.7 × 10^5^ V/m, the current density flowing through the mandrel is higher than that at the sheath, and the loss power of the mandrel is greater. Therefore, the temperature rise of the mandrel is higher than that of the sheath. A temperature difference is formed between the mandrel and the sheath, the heat is transmitted from the mandrel to the sheath. Similarly, in the case of the same current density, the area with high electric field strength will cause greater loss power. Therefore, the high-voltage side with the highest electric field strength [22] has the largest temperature rise.

In conclusion, the abnormal temperature rise of composite insulator #1 is caused by the deterioration of the mandrel. The deterioration of the mandrel leads to the value increase in its relative dielectric constant and tan*δ*. Then, the value increase in the relative dielectric constant and tan*δ* increases the current density flowing through the mandrel, and improves the dielectric loss power. Finally the loss power causes the abnormal temperature rise of the composite insulator. As the electrical parameter value at the deteriorated part of the mandrel changes greatly compared with that at the nondeteriorated part, the temperature-rise value caused by this defect is large, and the range usually extends from the hardware of the high-voltage side to several pieces of the umbrella skirt.

#### 3.3.2. Effect of Sheath Aging on Temperature Rise

**Figure 12:**
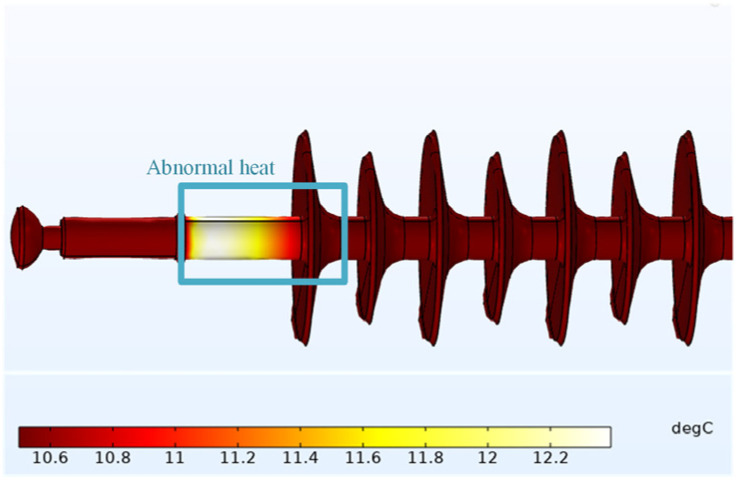
Simulation diagram of #2’s temperature rise.

Consistent with Section 3.3.1, assuming that both the ambient temperature and the composite insulator temperature at the initial time are 10 °C, the temperature distribution of #2 after 60 min is shown in Figure 12.

From the temperature-rise simulation diagram, it can be preliminarily seen that #2 only has obvious heating at the high-voltage side. The maximum surface temperature of the test object is 12.4 °C, and the temperature rise is 2.4 °C.

**Figure 13:**
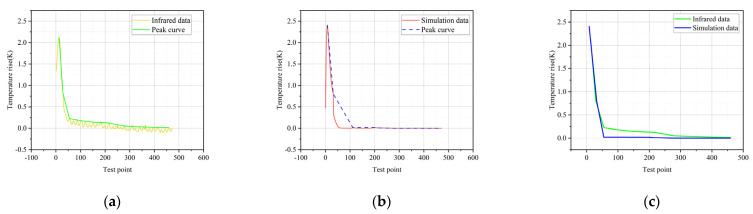
(**a**) Infrared temperature-rise curve of #2; (**b**) simulated temperature-rise curve of #2; (**c**) peak-curve comparison diagram of #2.

In order to verify the correctness of the simulation, the infrared spectrum is extracted and compared with the simulation results, as shown in Figure 13.

Figure 13a shows the temperature-rise data extracted from the infrared image in Figure 3b, and Figure 13b shows the temperature-rise data extracted from the simulation image in Figure 12. The temperature-rise curves in both figures start from the high-voltage side of the composite insulator, and the abscissa is the temperature test points along the axis of the insulator. It can be seen from the figure that the maximum temperature rise is at the high-voltage side, and the abnormal heating is only at the high-voltage side. Compare the peak curve of Figure 13a with that of Figure 13b, as shown in Figure 13c.

It can be seen that the temperature-rise curve of the composite insulator obtained by COMSOL is consistent with the actual situation. The abnormal temperature rise is only at the high-voltage side, and there is no obvious heating at other parts. The characteristics of the simulation results are completely consistent with the infrared. The Pearson correlation coefficient is 0.99555 and the *p*-value is 1.93 × 10^−8^, which indicates that the infrared data have a strong correlation with the simulation data. The accuracy of the simulation model is illustrated.

**Figure 14:**
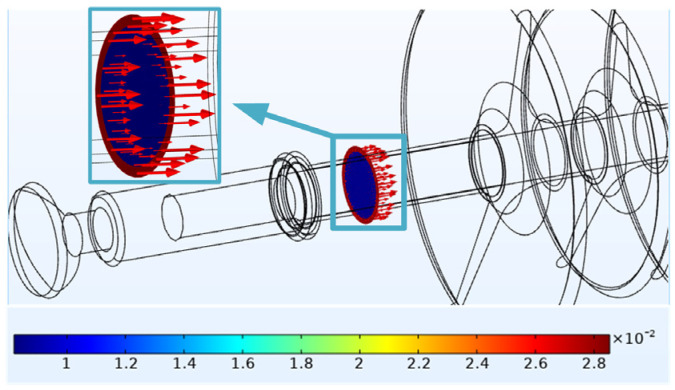
Current-density distribution of #2.

In order to explore the heating source of #2, the cross-sectional view of the high-voltage side with abnormal heating is further extracted for current-density analysis, as shown in Figure 14.

When t = 60min, the current density of the sheath is 2.8 × 10^−2^ A/m^2^, higher than the current density of the mandrel 8 × 10^−3^ A/m^2^. For #2, according to Formula (1), when the electric field strength is the same, the leakage current flowing through the sheath is greater than that of the mandrel; that is, the loss power at the sheath is greater than that at the mandrel. Therefore, the temperature-rise value of the sheath is greater than that of the mandrel. A temperature difference is formed between the mandrel and the sheath, and the heat is transmitted from the sheath to the mandrel. Similarly, when the current density is the same, the high-voltage side with high electric field strength has the maximum temperature rise. The reason why the temperature-rise value of #2 is smaller than that of #1 is that the current density of each part of #2 is smaller than that of #1’s mandrel.

In conclusion, the abnormal temperature rise of #2 is caused by sheath aging. After the sheath of the high-voltage side is aged, a large number of microdefects such as microcracks and micropores are formed on the surface. In the high-humidity environment, moisture invades the sheath microdefects, causing the increase in the dielectric constant and tan*δ*, thereby increasing the current density flowing through the sheath, and finally causing the abnormal temperature rise of the composite insulator. The temperature rise caused by this defect is small, due to the small change of electrical parameters at the aging part of the sheath. The abnormal temperature rise mainly occurs in a small range of sheath area, which is from the hardware of the high-voltage side to the first umbrella skirt.

## 4. Conclusions

In this paper, two composite insulators with different heating characteristics are tested and studied, and the two composite insulators are modeled by the finite-element-analysis simulation software COMSOL multiphysics. The following conclusions are obtained:

(1) The mandrel of test object #1 is deteriorated, the glass fiber is broken and the epoxy resin is oxidized and decomposed, while the mandrel of test object #2 has no obvious deterioration, the fiber is complete, and the fiber is connected with the fiber through epoxy resin, without obvious exposed fiber.
(2) For two composite insulators with abnormal heating, the temperature rise is mainly caused by dielectric loss. The increase in relative dielectric constant and tan*δ* will lead to the increase in current density, and then the change in current density will increase the loss power to cause an abnormal temperature rise.
(3) The electrical parameter value of #1’s mandrel is significantly higher than that of the normal part. The temperature rise caused by this defect is large, and the range usually extends from the hardware of the high-voltage side to several umbrella skirts; The electrical parameter value of the sheath at the high-voltage side of #2 is slightly higher than that at the normal position. The temperature-rise value caused by this defect is small, and the range usually appears in the sheath area from the hardware to the first umbrella skirt.
(4) By comparing the simulation results with the actual infrared images, the temperature-rise curve has the same trend, which verifies the rationality of the simulation model. The model can be used to distinguish the mandrel-deterioration defect from the sheath-aging defect, which has guiding significance and important reference value for the detection rate of mandrel deterioration and ensuring the safe and stable operation of the power grid.

## Figures and Tables

**Table 1:** Parameters of FXBW-220/100 composite insulator.

Model	FXBW-220/100
Height of structure (mm)	2350
Diameter of mandrel (mm)	24
Thickness of the sheath (mm)	3
Number of large umbrellas	23
Number of small umbrellas	22

**Table 2:** Thermal parameters of simulation model.

Materia	Thermal Conductivity/(W·(m·K)^−1^)	Specific Heat Capacity/(J·(kg·K)^−1^)	Density/(kg·m^−3^)
Air (75% humidity)	0.0011	1.01	25
Silicon rubber	0.27	1700	1100
Mandrel	0.4	535	2000

**Table 3:** Electrical parameters of composite insulator.

Test Object	Silicone Rubber		GFRP	
	Relative Permittivity	tan*δ*	Relative Permittivity	tan*δ*
#1’s Heated part	7.35	13.2%	26.73	52.1%
#1’s Nonheated part	5.52	4.1%	5.41	2.2%
#2’s Heated part	6.15	8.2%	5.41	2.2%
#2’s Nonheated part	6.15	8.2%	5.41	2.2%

## Data Availability

The data presented in this study are available on request from the first authors and corresponding author.
